# Indents

**Published:** 1922-11

**Authors:** 


### INDENTS

**☞ Brief Articles of Technical or Scientific Value will receive notice and comment. Contributions invited.**

---

Treating An Exposed Pulp. To treat an exposed pulp for a lower first permanent molar, place a crystal of thymol on the point of exposure and then touch it with a hot instrument; this will cause the thymol to flow over the exposure and recrystalize it, then cover it with a thick paste of zinc oxide and eugenol. Cover the exposure with any hard cement.—Charles A. Sweet, in *Pacific Dental Gazette,* September, 1922.

---

Honors in Dentistry. In this age of commercialistic strenuosity we are prone to lose sight of the things that make life and effort worth while. We are easily engrossed in the struggle to provide properly for those dependent on us and in addition to lay aside a competence for the non-productive days. This, of course, is the first law of nature and our first obligation to those we love, but next comes our duty to our profession, a duty that can not be discharged in dollars and cents, but one which should be fulfilled in such a manner as will repay our obligation to the profession and at the same time strengthen the foundations upon which our forefathers have builded.

And the greatest return or reward, if we wish to view it in that light, is in the satisfaction of having done our duty. But there are other rewards for professional effort and endeavor which count for much in that they constitute a visible expression of appreciation of our effort by those about us.

No man should be actuated to his professional duty by the hope of ultimate reward other than in the personal satisfaction he derives from the knowledge of work well done, but all of us are human enough to desire an outward show or expression of approval from our fellowmen.

The profession is well acquainted with the history and purpose of the Jarvie medal which is awarded each year by the New York Dental Society to a dentist who has rendered distinguished service to his profession. This is undoubtedly one of the most gracious honors bestowed by dentistry, and the professional ambition of any dental practitioner should be fully gratified to be grouped with the distinguished list of those who have received this medal in the course of the last nineteen years.

Imbued with the same spirit that actuated the establishment of the Jarvie medal, Mr. Leonard A. Jenkins, son of the late Dr. Newell Sill Jenkins, has been impelled to establish a similar memorial to his father, and accordingly a fund has been established and placed in charge of the Connecticut State Dental Society, from the proceeds of which a medal will be awarded each year to someone in dentistry or in the arts and sciences who has performed notable service to humanity.

At the meeting in April this year the first award was made, and no more fitting subject could have been chosen than the choice of the committee of the society to whom the award was given. Dr. Alfred C. Fones, of Bridgeport, Conn., was the recipient of the first award, and all who are familiar with the work Dr. Fones has done during the past decade readily acquiesce in the wisdom of the choice.

Dr. Jenkins' professional career was dominated by his interest in his profession and the welfare of humanity; the keynote of his life was his love of humanity and his every professional effort was directed to human welfare. The predominant characteristic of the man for whom this memorial has been established was his love of humanity, his courteous kindliness and his abiding faith in the goodness of mankind, which was but an outward expression of his own nature. These characteristics impelled him to take an active part in the International Dental Federation, where his efforts were directed to the advancement of oral hygiene propaganda for the benefit of the children of the world.

It is thus quite fitting that the first award of a commemorative medal of Newell Sill Jenkins should go to one whose work has meant and in the future will mean untold benefits to the children of the land.

The Connecticut Society has done well to follow the lead of the New York Society in thus commemorating the memory of a beloved member of the dental profession, and has followed an example that it would be well for as many societies as can to emulate. Too few of the real honors of life come to the toilers, and more such benedictions would redound to the glory and honor of dentistry.

On one occasion when the Jarvie medal was awarded to a distinguished research worker the recipient remarked that he had devoted a large part of his time for forty years and had spent much of his income in the investigation of the many problems of dentistry, but that this was the first public recognition his efforts had received from the dental profession. This is a sad commentary on the generosity of a humane profession and one that such benefactions as we have above noted will make improbable in the future.

Few men would have the persistence to spend years of their life in a work that encroached largely on their income as well as time, without some reward or recognition from the profession, and there are too few men in the profession fitted and willing to pursue such work for such efforts to go unrecognized.

Jenkins was not a man who worked for or thought of reward, nor is it true that most of our researchers and benefactors have any hope of reward in mind, but as long as human nature is as it is, worth-while honors will go far to encourage, if not stimulate, men to noble efforts even in a profession whose very *motif* is humanitarianism.—*Dental Cosmos.*

